# Glenosphere total disengagement secondary to security screw breakage in reverse total shoulder arthroplasty: two case reports

**DOI:** 10.1016/j.xrrt.2026.100814

**Published:** 2026-07-08

**Authors:** Saad Alhajraf, Moyad Awad, Ryan Bicknell, Andrew Trenholm

**Affiliations:** a Dalhousie University, QEII HSC - HI Site, Halifax, Nova Scotia; b Queen's University, Kingston General Hospital, Kingston, Ontario; c Dalhousie University, QEII HSC - HI Site, Halifax, Nova Scotia

## Introduction

The indications for reverse total shoulder arthroplasty (rTSA) have continued to expand since its approval by the Food and Drug Administration in 2004.^3^ Current indications for rTSA include cuff tear arthropathy, massive rotator cuff tears without osteoarthritis, primary osteoarthritis, inflammatory arthritis, revision arthroplasty, proximal humerus fracture, and tumor resection.^3^ With this expansion in indications, the number of rTSAs done worldwide has significantly increased over the past 2 decades.^6^ Despite the multiple reports of pain relief and improved patient function and satisfaction, the complication rate for rTSA is still considered relatively high, ranging from 20% for primary surgeries and 40% for revisions.^5^ Reported complications include, but are not limited to, infection, scapular notching, instability, glenoid loosening, acromial fracture, and periprosthetic fractures.^10^ Few studies have reported glenosphere disengagement or complete dissociation from the glenoid baseplate due to its rare occurrence.^4^ The early Grammont's rTSA designs had peripheral threads around the glenoid baseplate into which the glenosphere would screw. The glenosphere tended to unscrew. Then the design changed in 1996 to a peripheral Morse-taper locking mechanism reinforced by a countersunk screw.^11^ In 2008, Middernacht^8^ published the first review of glenosphere disengagement in 14 cases, 3 of which had a complete dissociation secondary to central security screw failure.

In this article, we review 2 cases of glenosphere dissociation secondary to security screw failure; no previous cases using similar implants (Delta Xtend Reverse implant [DePuy Synthes]) have been reported. Consent was provided by both patients, who were informed that the data related to the case were collected to be submitted for publication.

## Case 1

**Figure 1:**
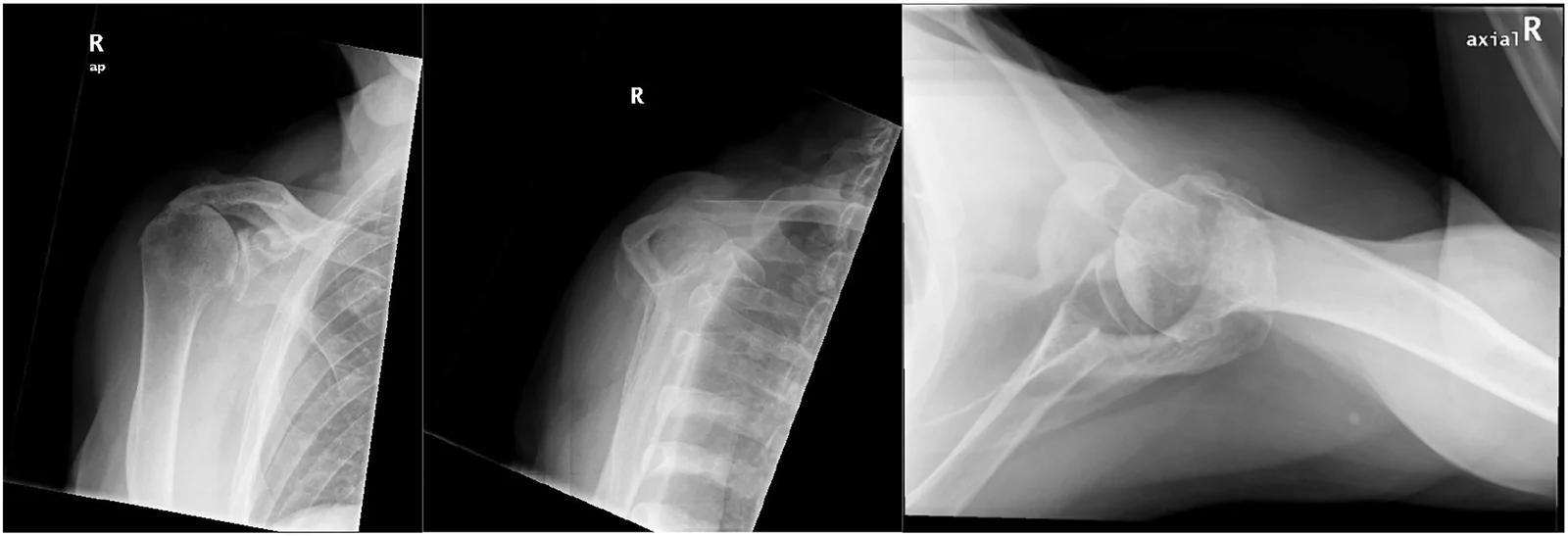
Case 1 pre-operative x-rays showing severe cuff tear arthropathy.

**Figure 2:**
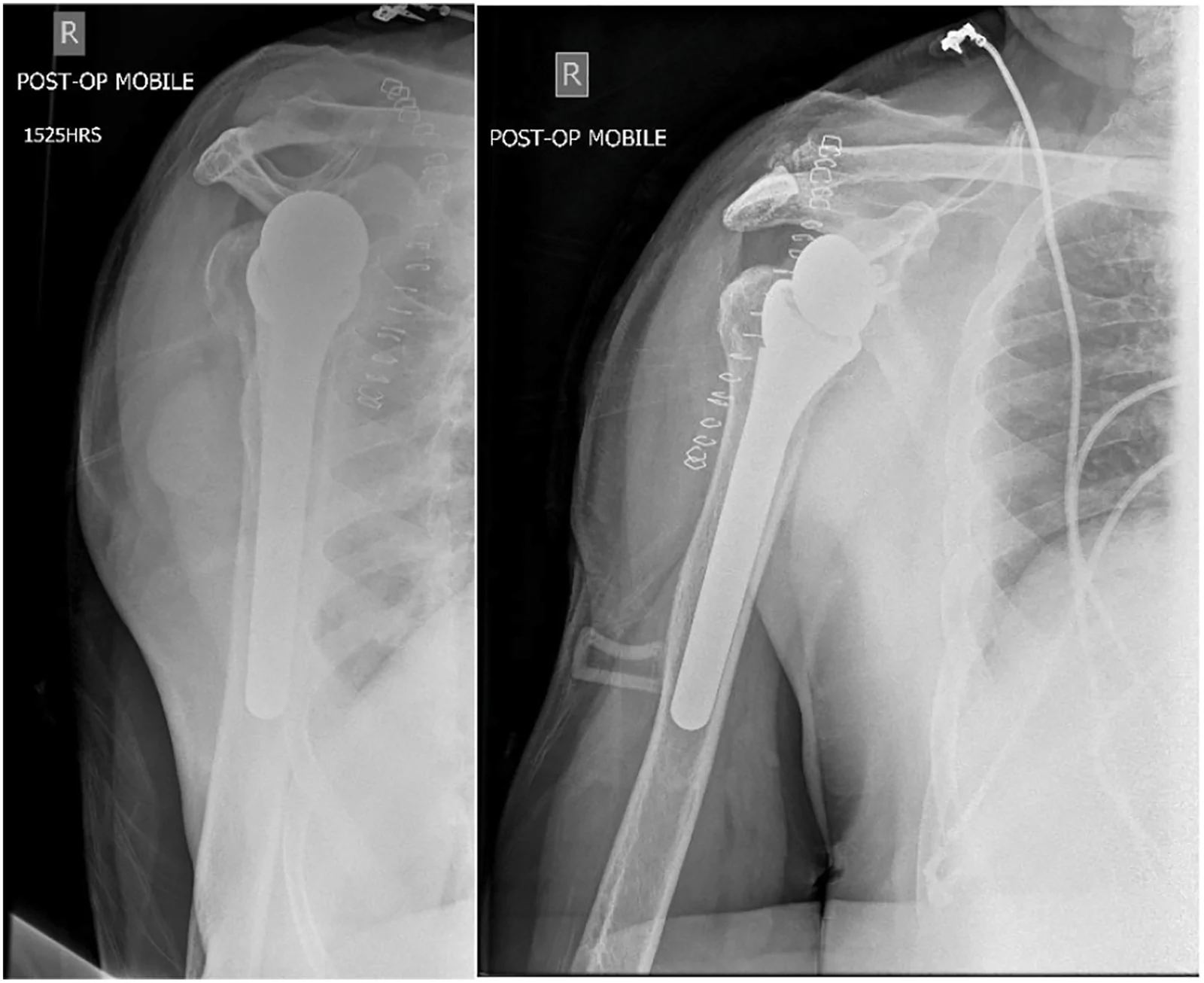
Case 1 immediate post-operative x-rays.

**Figure 3:**
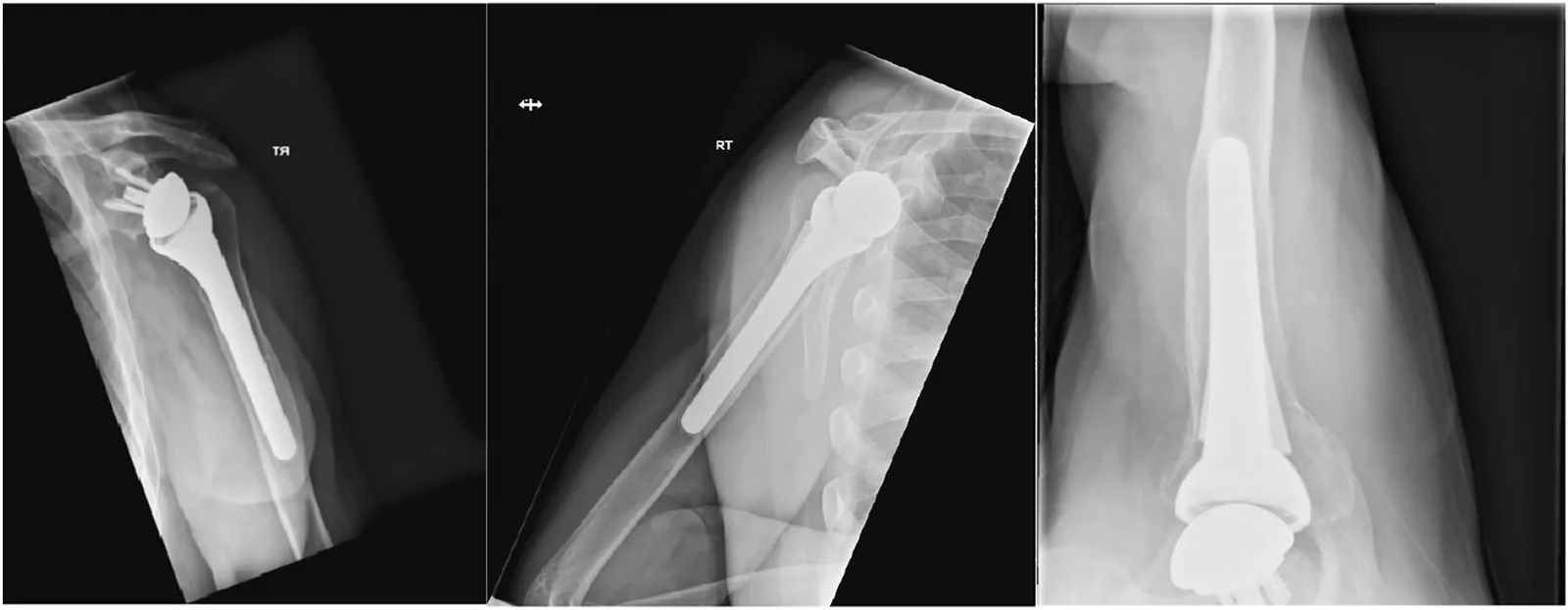
Case 1 x-rays at a 2-year follow-up visit.

**Figure 4:**
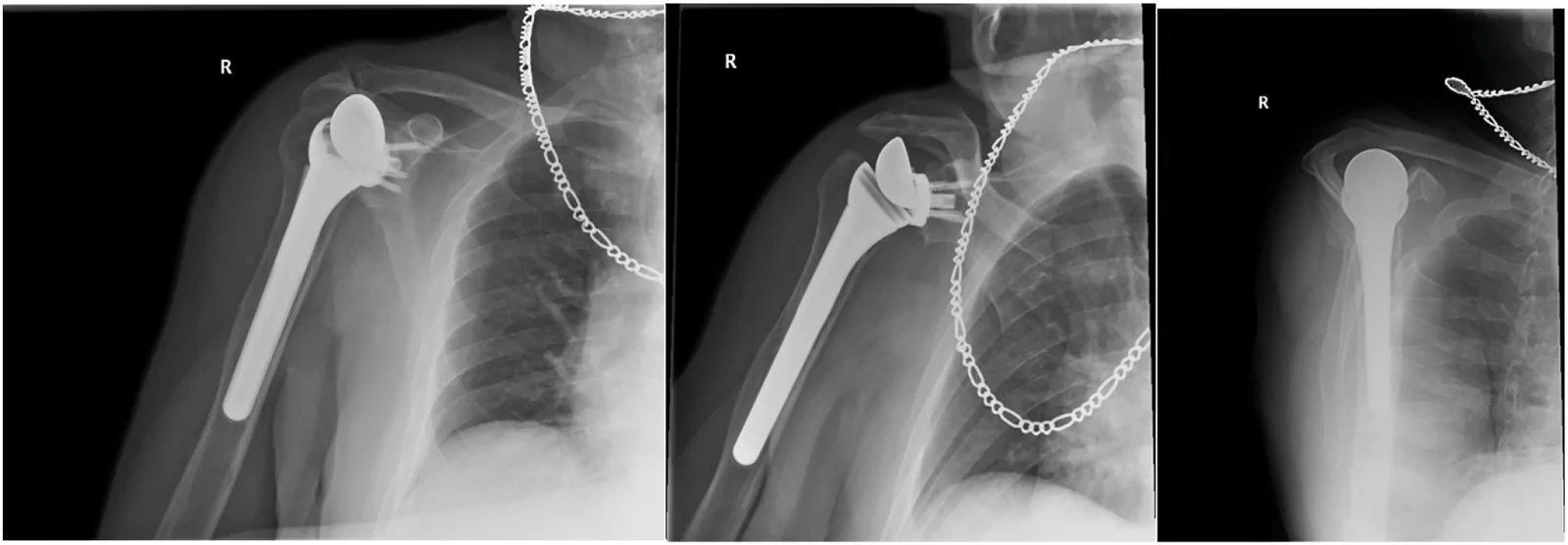
Case 1 x-rays show screw breakage and glenosphere total disengagement.

A 75-year-old white male presented to the orthopedic clinic after a referral was made from his family doctor regarding progressive right shoulder pain. He is a right-hand dominant retired dairy farmer. When he was first seen in the clinic, his main complaint was bilateral shoulder pain, worse on the right side, associated with a significant decrease in function, especially with overhead activities in the same shoulder. There was no previous trauma to the shoulder that he could recall. His pain was well tolerated at night and did not affect his sleep. Otherwise, he was a healthy, nonsmoker, not on any medications, and had no known drug allergy. On physical examination of the right shoulder, there was no significant wasting and no tenderness with palpation around his shoulder. There were no signs of axillary nerve palsy. His range of motion (ROM) was minimal (pseudoparalysis), with active motion of 70° on forward elevation and passive motion up to 120° with pain; his external rotation was limited to 20°. Internal rotation strength was preserved, and abduction and external rotation were 4/5 compared to the contralateral side. Looking at his x-ray of the right shoulder showed end-stage cuff tear arthropathy with proximal humeral head migration and acetabulization of the acromion (Hamada grade III) (Fig. 1). Ultrasound was done and showed a massive rotator cuff tear. After discussing the options with the patient, the decision was made to proceed with a right reverse total shoulder arthroplasty (rTSA) and a computed tomography scan for surgical planning. The surgical procedure was uneventful with a DePuy Delta Xtend reverse shoulder arthroplasty system, including a size 38 glenosphere (Fig. 2), and the patient was discharged on post-operative day one with an arm sling and instructions for his ROM limitations. The patient was seen 2 weeks later, and his staples were removed without any wound complications or signs of infection. At the 6-week mark, formal physiotherapy was started as per the treating surgeon protocol, and his ROM improved to 120 degrees of forward elevation and abduction, 0 degrees of external rotation, and internal rotation to L5. One year after the surgery, the patient was seen again. At the time, he was doing remarkably well with a ROM of 160 degrees of abduction and forward elevation, external rotation of 10°, and internal rotation to L5 and back to his daily activities without any difficulties. He was given a follow-up at the 2-year mark from his surgery. At the 2-year follow-up, the patient began to complain about a new sensation of implant dislocation that he was able to self-reduce at home. These events happened around 3-4 times over the course of 1 month and were accompanied by severe pain that immediately resolved once the shoulder was reduced. No specific movement was noted to produce these episodes, and he usually reduced the shoulder with abduction and the help of his wife. The X-rays performed on that visit did not show any signs of instability or implant failure (Fig. 3). During the physical exam, he had full, smooth ROM with no signs of apprehension, and no swelling or erythema was noted around the incision or the shoulder overall. The plan was to start with blood work and a bone scan to rule out infection and implant loosening. The results revealed regular inflammatory markers and a moderate uptake on bone scan around the implant of unclear significance, but it was considered to be abnormal given that it was done 2 years after surgery. Stable alignment of the implant was noted on the x-rays. One week after his bone scan, he presented to an outside hospital with a similar dislocation episode, but this time, he was unable to self-reduce it at home, and his x-ray showed a complete disengagement of his glenosphere from his glenoid baseplate (Fig. 4). There were no signs of axillary nerve palsy. The patient was transferred to our hospital, and after discussing the options with him, the decision was made to proceed with a revision rTSA.

### Surgical procedure

**Figure 5:**
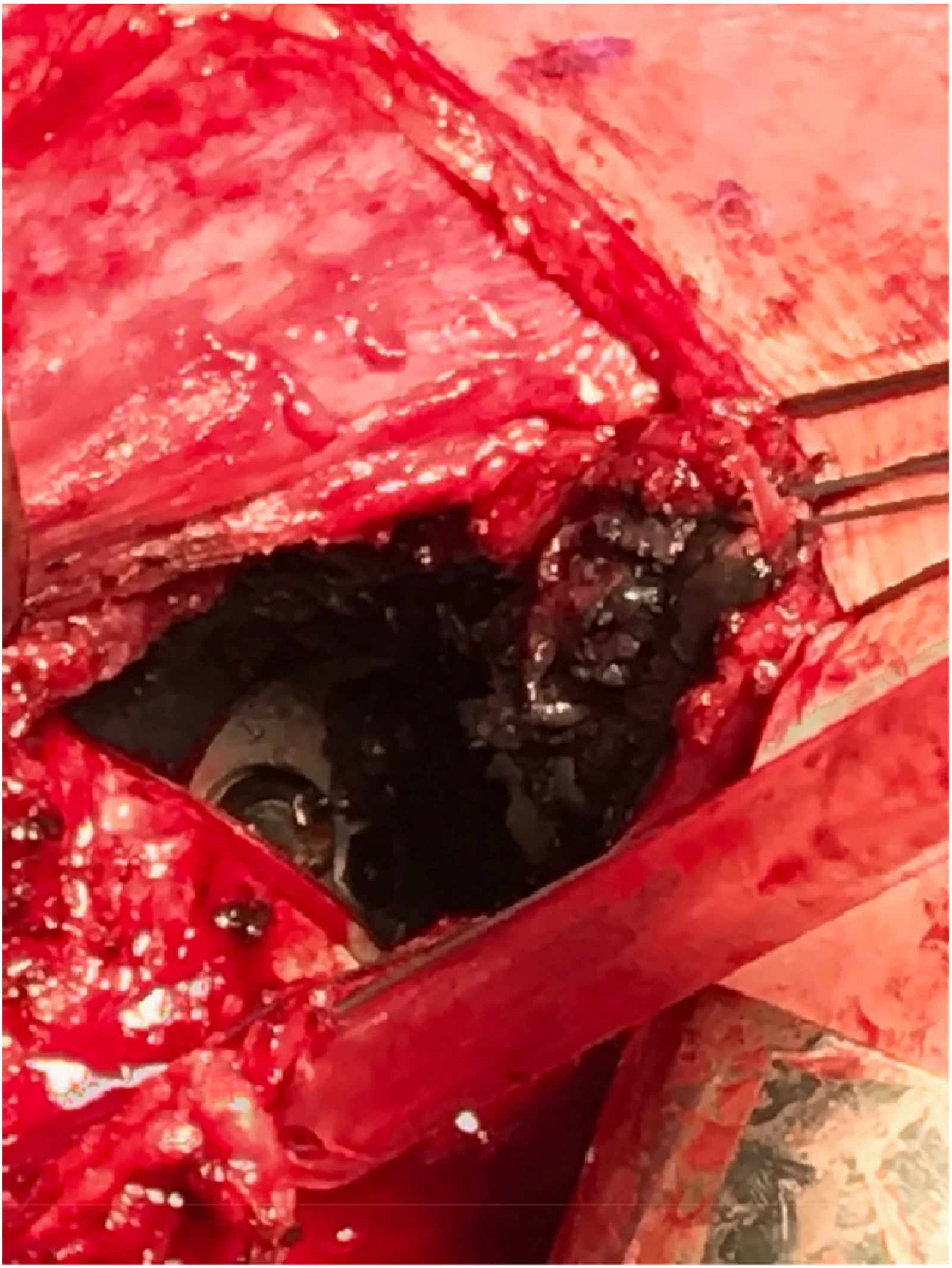
Extensive peri-implant metallosis involving the subscapularis muscle.

**Figure 6:**
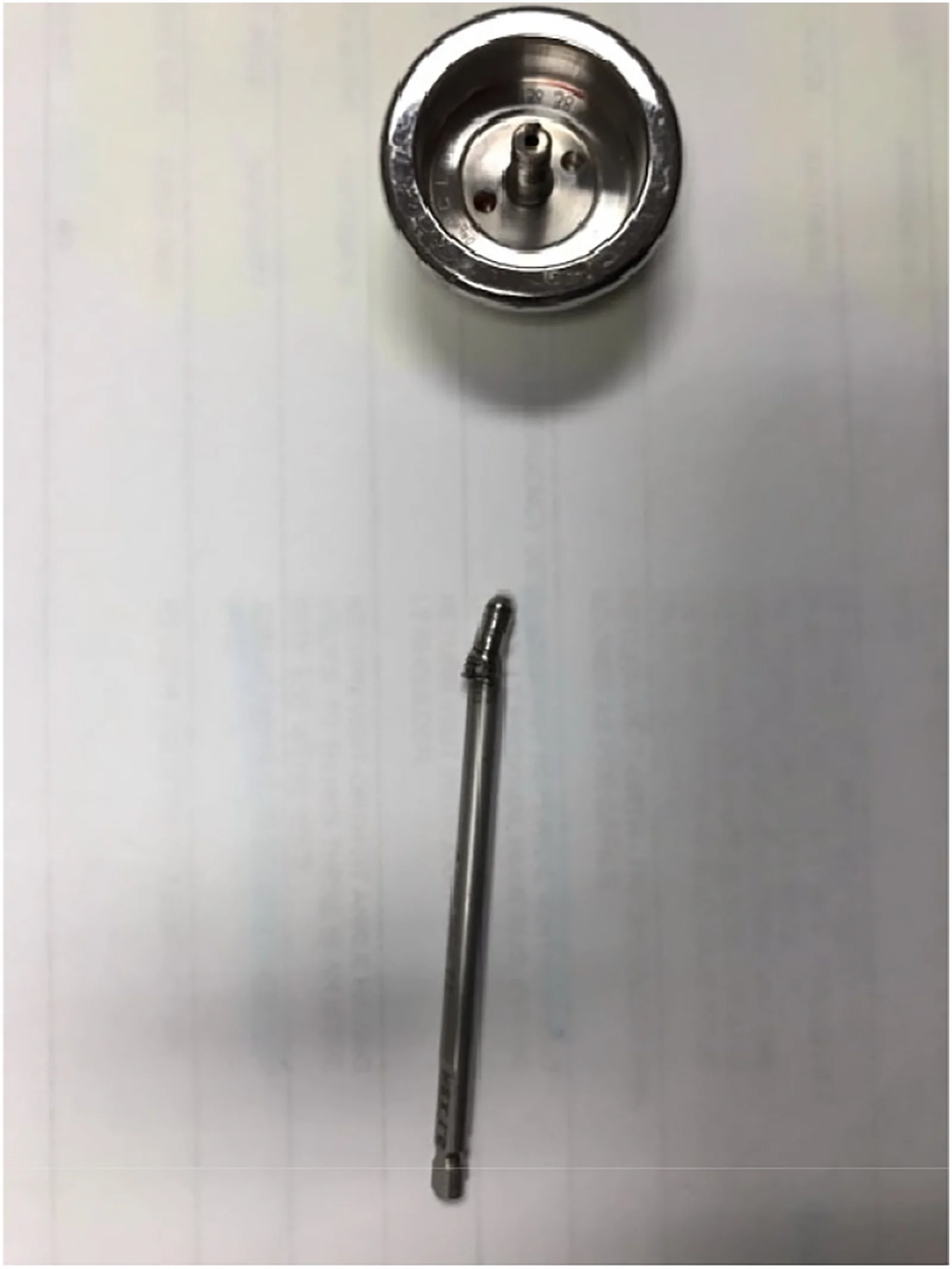
Picture showing the broken glenosphere screw leading to disengagement.

Surgery was performed under general anesthesia in the beach chair position. The previous incision, which was a standard deltopectoral approach, was utilized. Once the subscapularis was released, significant and extensive metallosis was seen (Fig. 5), with no obvious evidence of infection. Intraoperative cultures were taken both deep and superficial and sent for analysis. After releasing the subscapularis muscle, we were able to see the fully disengaged glenosphere. The central security screw was broken between the baseplate and the glenosphere (Fig. 6). With the use of the broken screw removal instruments, we were able to drill into the remaining screw and disengage the threads and remove the screw without having to remove the baseplate. The metaglene baseplate was stable and solid, so it was not removed. In the primary procedure, we used a 10-degree tilt guide. The inclination and version of the metaglene were both adequate in the revision procedure. We think the main reason for the failure was the locking mechanism. The glenosphere was changed to the same size, 38, but eccentric, in case he was notching. The polyethylene humeral insert was exchanged with the same size. The humeral stem was stable, so it was not revised.

### Post-operative recovery

**Figure 7:**
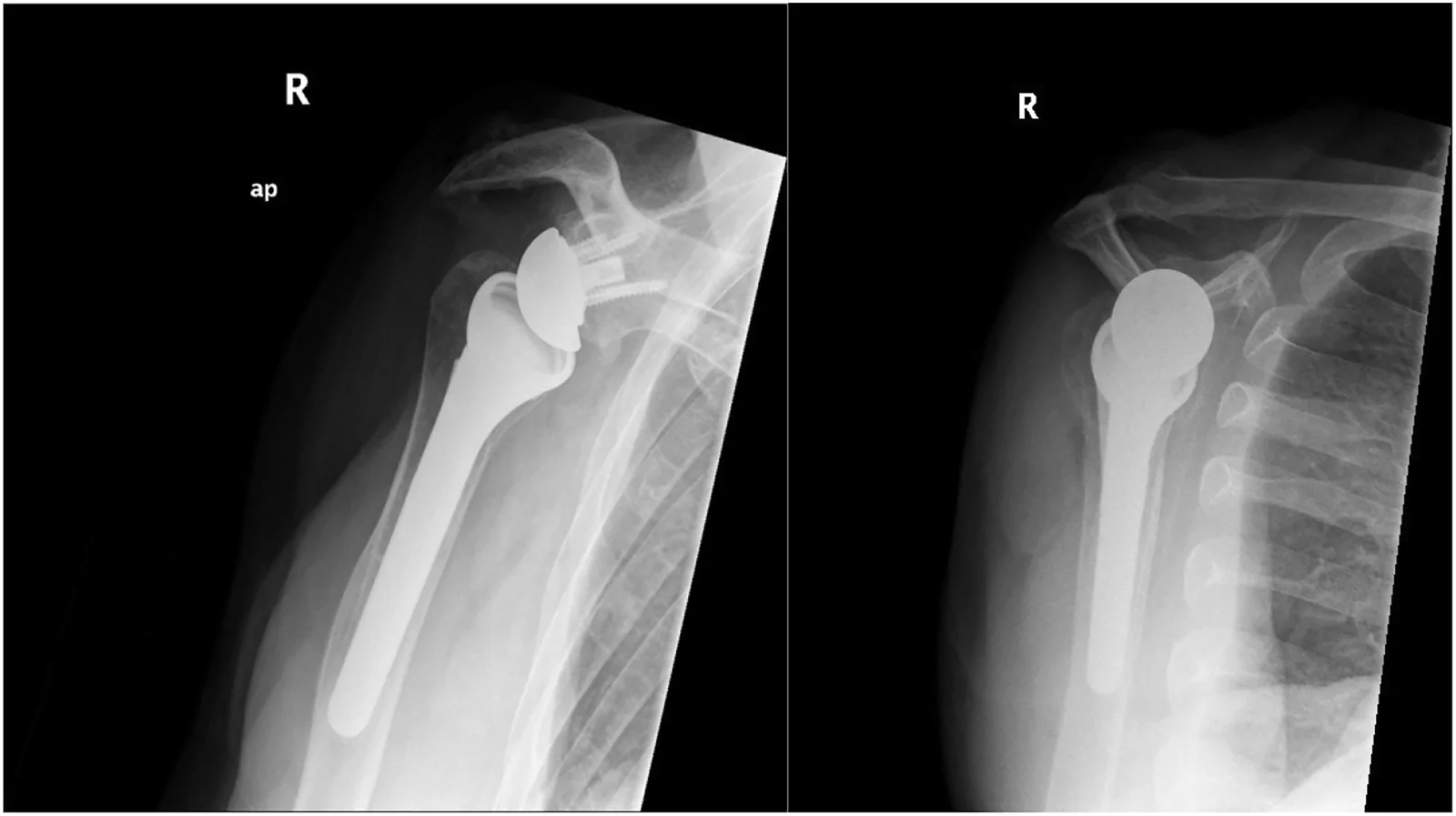
X-rays at 2-year follow-ups show stable implants without any signs of complications.

The patient was discharged from the hospital on post-operative day 1, with instructions to remain in a sling for 6 weeks with only ROM exercises for the elbow and wrist. After 6 weeks, start physiotherapy according to our standard rehabilitation protocol. After 6 weeks, only permanent restrictions are 25-30lb lifetime repetitive lifting restriction. No overhead throwing sports or contact sports. At the 2-year mark, a functional ROM was achieved, reaching a forward elevation of 110°, abduction of 110°, external rotation of 30°, and internal rotation up to L5. He returned to his normal daily activities without difficulty. He has no pain. He has no clicking or sensation of instability. His x-ray showed a stable implant without any signs of complications (Fig. 7).

## Case 2

**Figure 8:**
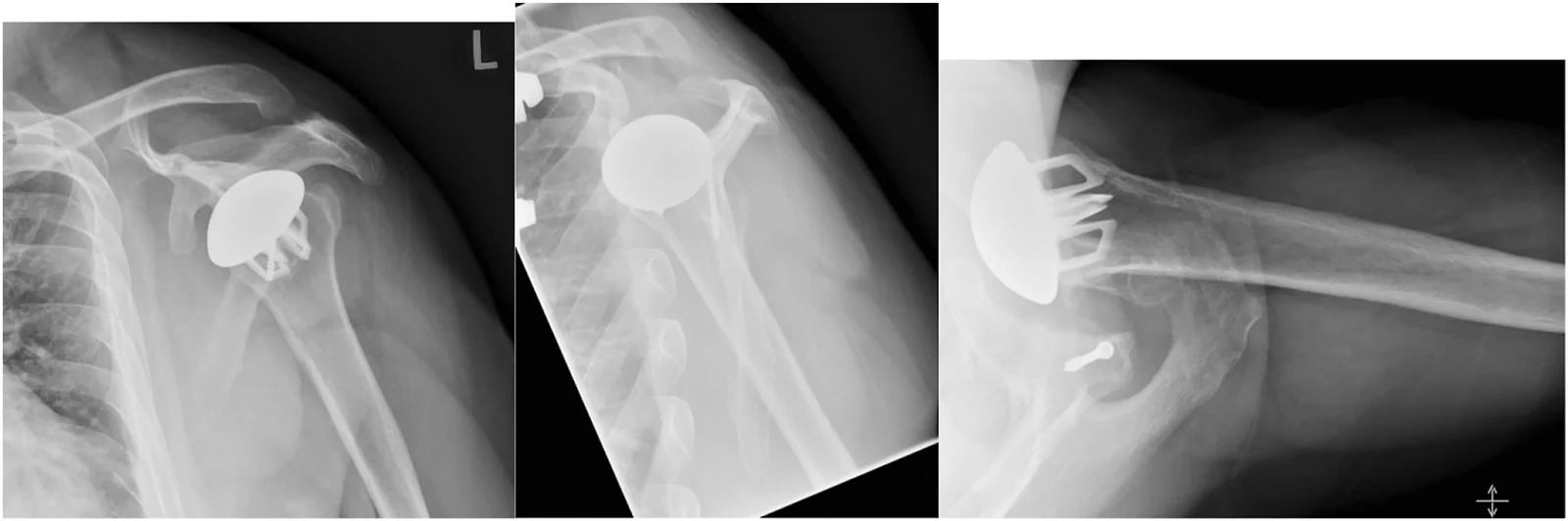
Case 2 pre-operative x-rays showing anterosuperior escape of a previous shoulder hemiarthroplasty.

**Figure 9:**
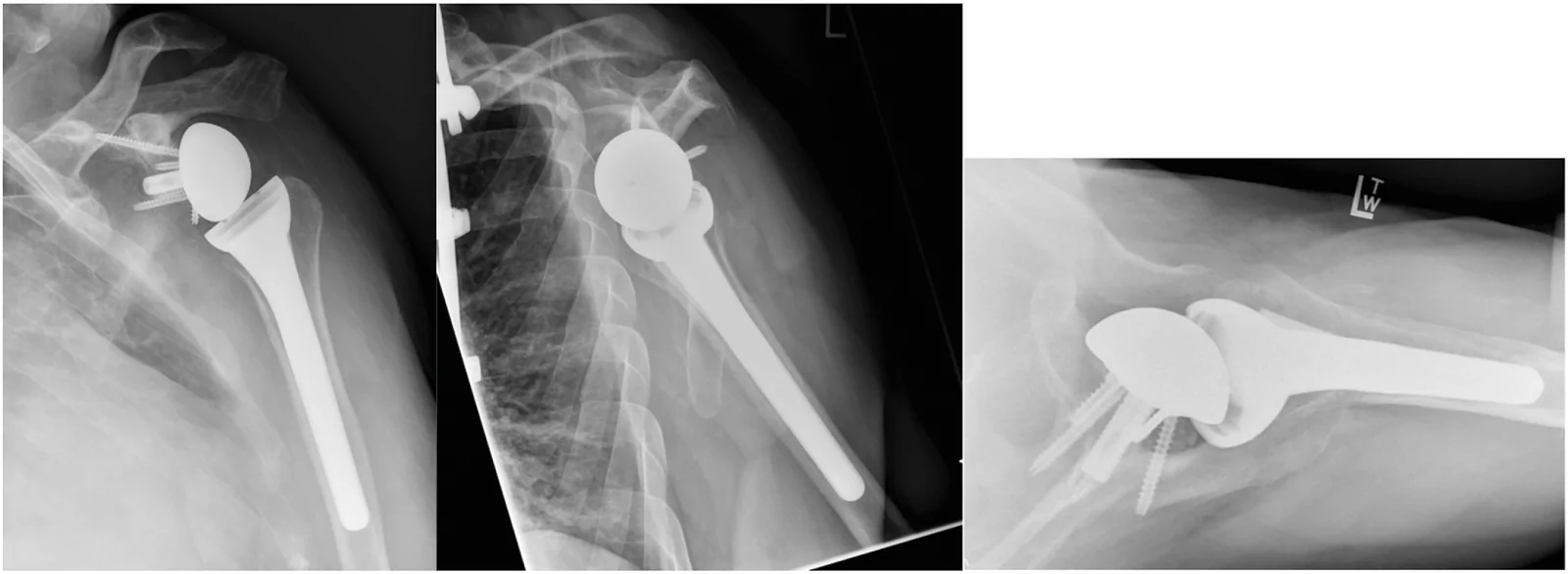
Case 2 post-operative x-rays.

A 50-year-old male patient presented initially with a left shoulder dislocation with glenoid, acromion, and more significant tuberosity fractures that were all treated with open reduction and internal fixation. This initial injury was 4 and a half years prior to the rTSA procedure. He underwent a hardware removal and left shoulder hemiarthroplasty 2 years later for post-traumatic arthritis and then the left rTSA 3 years after that for anterosuperior escape (Fig. 8). At no point following any of his previous procedures was there any concern with infection, either clinically, radiographically, or based on laboratory investigations. The rTSA surgical procedure was relatively uneventful with a DePuy Delta Xtend rTSA system, including a +10 mm long stem glenoid baseplate and a 42-centered glenosphere (Fig. 9).

**Figure 10:**
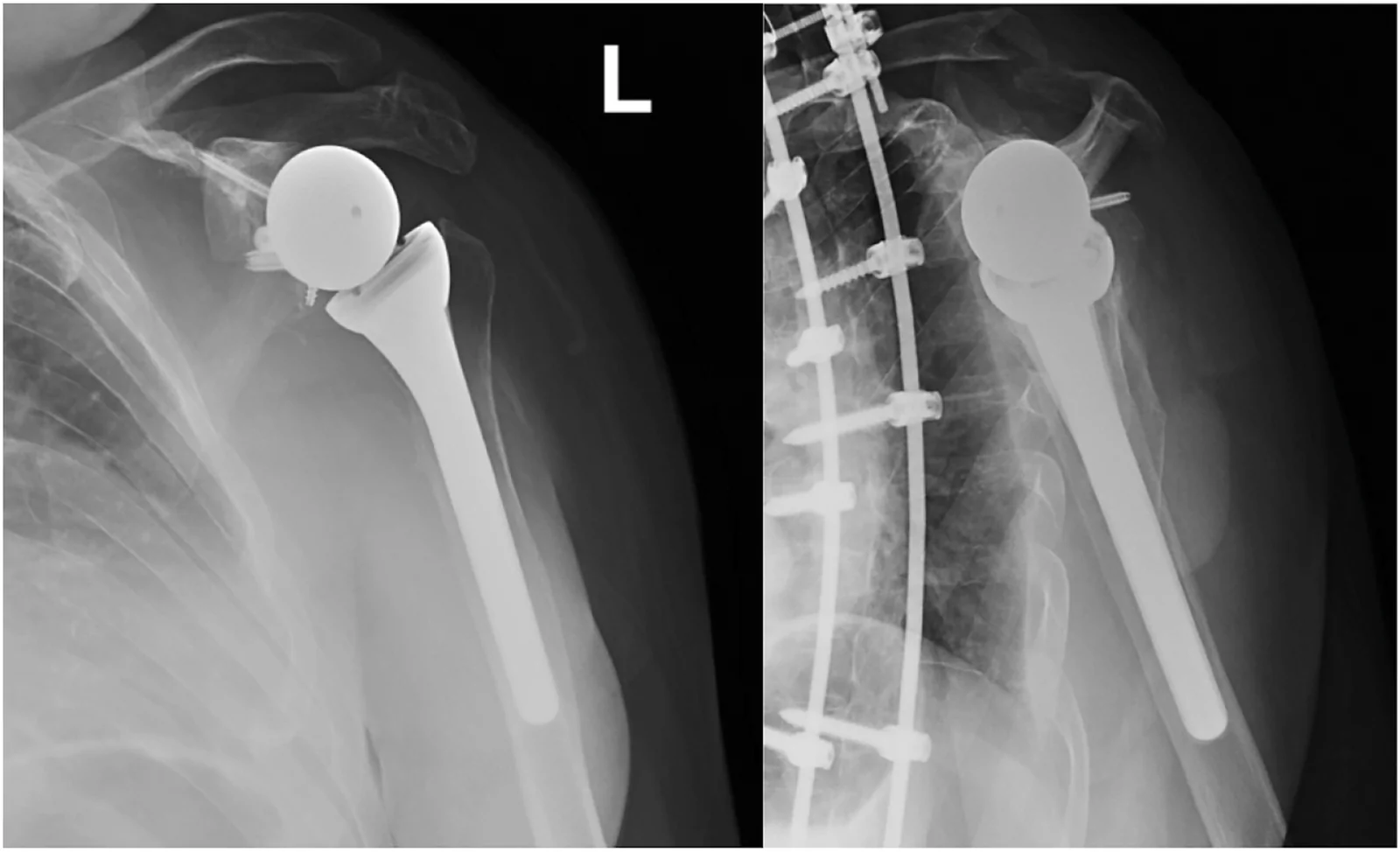
Case 2 x-rays at 3-year follow-up.

**Figure 11:**
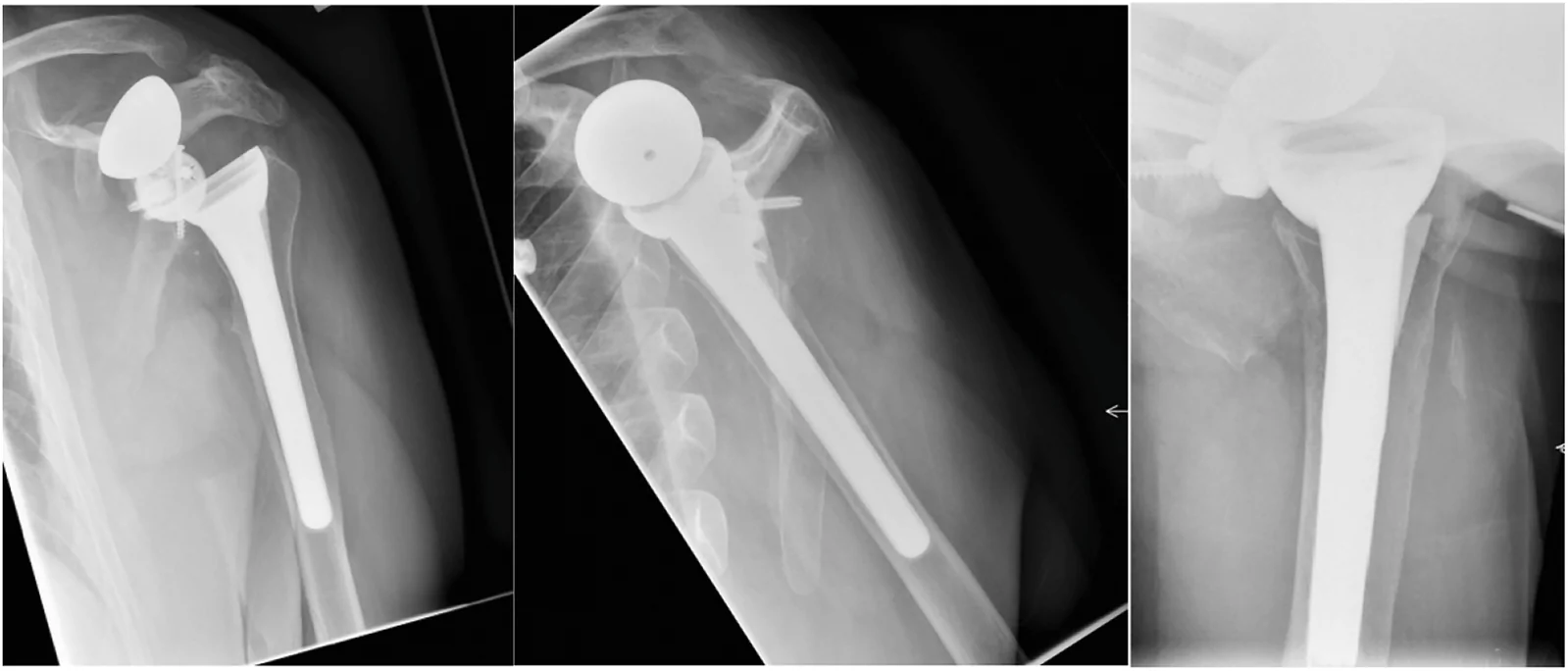
Case 2 x-rays showing complete glenosphere disengagement.

He did reasonably well after his rTSA and was followed clinically and radiographically every year. At his routine follow-up, almost 3 years post-operatively, he had some intermittent pain with activity but was tolerable and much improved from pre-operative. His incisions were all well-healed, but he had persistent atrophy of his anterior deltoid. His active ROM was forward flexion of 100°, abduction of 90°, external rotation of 20°, and internal rotation in abduction of 30°. Radiographically, he had appropriate alignment with no evidence of loosening or scapular notching (Fig. 10). He then presented a year and a half later with a 6-month history of clicking in his shoulder with movement and a progressive loss of movement over that time. This was not associated with any traumatic incident. He denied any worsening of his pre-existing intermittent pain. An x-ray showed a complete disengagement of his glenosphere from his glenoid baseplate (Fig. 11). There were no signs of axillary nerve palsy. After discussing the options with him, he agreed to go for revision rTSA.

### Surgical procedure

**Figure 12:**
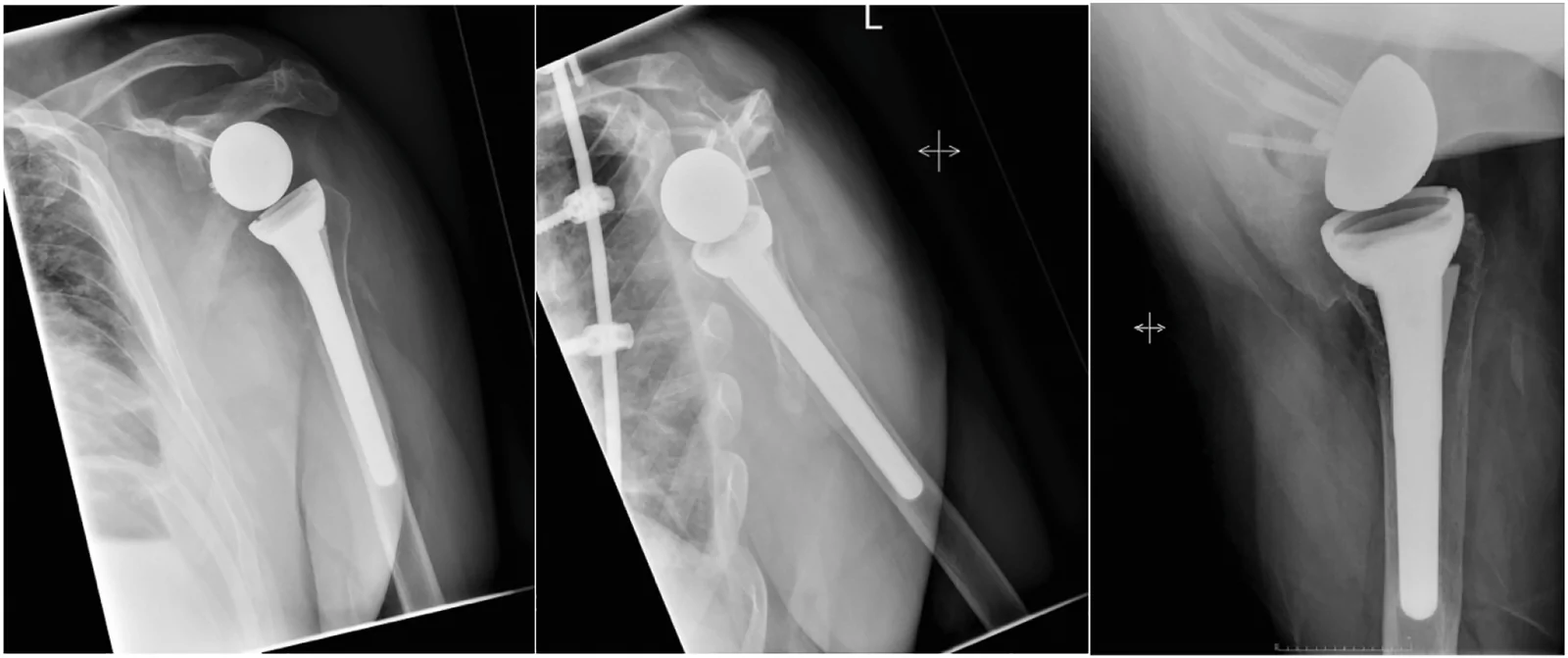
Case 2 post revision x-rays.

Surgery was performed under general anesthesia in the beach chair position. The previous deltopectoral incision and a standard deltopectoral approach were utilized. There was no subscapularis present and no evidence of infection. Intra-operative cultures were taken, both deep and superficial, and eventually, they did not show any evidence of infection. We were able to see the fully disengaged glenosphere. The central security screw was broken between the baseplate and the glenosphere. With the use of the broken screw removal instruments, we were able to drill into the remaining screw and disengage the threads and remove the screw without having to remove the baseplate. The baseplate was solid, so it was not removed. Both the glenosphere and the polyethylene humeral insert were exchanged with the same size. The baseplate and humeral stem were stable, so they were not revised (Fig. 12).

Similar to case 1, we thought the problem was a failure of the locking mechanism, and inclination or version would not affect it, as they were adequate intraoperatively. We did not change the glenosphere size, as it remained stable with the reduction. We think the main reason was a failure of the locking mechanism, and we thought changing the size would not affect it.

### Post-operative recovery

The patient was discharged from the hospital on post-operative day 1, with instructions to remain in a sling for 6 weeks and then start physiotherapy according to our standard rehabilitation protocol. The patient has temporary lifting restrictions of less than 5 pounds for the first 3 months. Then no lifetime restrictions. At a year post-op, the patient felt he had returned to his baseline. At his last follow-up, 2.5 years after the revision surgery, he continued to complain of intermittent pain with activity, similar to his baseline, prior to his revision surgery. He has no clicking or sensation of instability. He has 60 degrees of active forward elevation and 90° passively. Abduction is 45° actively and 90° passively. Internal rotation is to the hip, and he has 0° active and 20° passive external rotation.

## Discussion

In our center, a total of 1545 shoulder arthroplasties were done since 2005. We have 2 upper extremity surgeons who are doing shoulder arthroplasties. These are the only 2 cases that we identified with glenosphere disengagement. Most of the current rTSA designs share a common Morse-taper feature in which the glenosphere will lock onto the baseplate after making sure it is well seated intraoperatively. Since this modular connection is in a biological environment and is subjected to mechanical stress during functional activities, the interface will be exposed to mechanical wear, corrosion, chemical degradation, fatigue, and eventually failure in the form of disengagement.^2^ The first glenosphere dissociation or disengagement report was documented by Middernacht in 2008.^8^ In his review of 479 rTSAs. They found 16 cases of disengagement. Of these 16, only 3 (Delta III, DePuy) had complete disengagement due to central screw failure. He suggested in his review that soft tissue interposition, inadequate reaming, and unfamiliarity with the central screw locking mechanism were possible causes for this mode of failure. In 2015, Fuller^4^ reported a similar case of central screw failure in a non-Morse-taper implant (Equinoxe, Exactech, Gainesville, FL, USA). In his review, he reported 2 cases of disengagement; one of them was a complete failure of the central screw that led to full dissociation and required revision surgery. The second case had only partial disengagement on x-ray, but due to the concern of having a more difficult revision surgery if the screw was broken, the decision was made to proceed with revision. They hypothesized that the failures happened for 2 possible reasons. First, the angle of the central screw in their system is not central on the glenosphere, so when trying to line up the screw perpendicular to the glenosphere, this may lead to entering the baseplate off-angle and will lead to cross-threading. Second, the baseplate has a bone cage that allows bony ingrowth to the glenoid, but at the same time, the holes on the cage may prevent full seating or interlocking between the screw and the baseplate threads. More recently, Ramirez^9^ reported a similar case (Equinoxe, Exactech) 2 years after surgery. In his case, there were no radiological signs of screw breakage, and it was only found intraoperatively. Cusick et al^2^ reviewed all of the cases that were done by the senior author of the article and found only 13 cases (reverse total shoulder prosthesis RSP, DJO) of dissociation. They had 2 other cases that were done outside of their facility (Trabecular Metal reverse shoulder system, Zimmer). The 2 implants differ in that the RSP has a central locking screw, while the Zimmer rTSA relies only on the Morse taper locking mechanism. In their review, they found that dissociation was significantly associated with bigger glenosphere sizes (>40 mm). Five implants were available for analysis after retrieval, and based on the Modified Goldberg Score,^9^ 4 out of 5 implants received a score of 1, which indicates minimal fretting on <10% of the surface and no corrosion. In their biomechanical evaluation, they concluded that improper taper engagement reduced the torsional capacity at the interface by 60%. Our review found that most of the disengagement occurred from 6 months to 2 years post-operatively, with improper intraoperative seating of the glenosphere being the main reason for failure.

In our review, case 1 had a unique presentation of the recurrent feeling of dislocations without any radiographic evidence of failure, which has not been reported in the previous literature. A similar presentation should raise the surgeon's suspicion of a glenosphere failure in those challenging cases, as, most likely, those multiple dislocations or motions lead to the screw breakage. We recommend carefully considering the tactile and auditory feedback while engaging the glenosphere to the baseplate. The baseplate and glenosphere should be completely dry before impaction. Blevins^1^ showed that even 0.4 mL of water or blood could prevent full engagement of the Morse-taper and contamination of the taper socket can decrease the force required to disengage the taper by 24.3% with water and up to 76.9% with blood. Surgeons should be familiar with the implant's locking mechanism to ensure proper seating. Managing such a complication can be difficult if it is associated with baseplate loosening due to the significant bone loss in the glenoid. Gladly, all previously reported cases did not require baseplate revision, and glenosphere exchange was enough. Overall, we think that there are 3 different types of glenosphere disengagement. Type I is a partial disengagement on x-ray in an asymptomatic patient. Type II is a partial disengagement on x-ray associated with symptoms of instability or pain. Type III is a total disengagement between the glenosphere and the baseplate. We recommend close follow-up with repeated radiograph for type I, as there is a possibility of spontaneous reassembly.^7^^,^^8^ For type II and III, surgical revision with glenosphere exchange is recommended. We summarize them in Table I.

**Table I:** Classification of glenosphere disengagement after reverse total shoulder arthroplasty: radiographic findings, symptoms, and management.

Type	X-ray	Symptoms	Management
Type I	Partial disengagement	Asymptomatic	Close follow-up.^6^^,^^7^
Type II	Partial disengagement	Instability or pain	Glenosphere revision
Type III	Total disengagement	Instability or pain	Glenosphere revision

## Conclusion

Glenosphere dissociation is an infrequent complication after rTSA. This complication was thought to occur in early rTSA designs due to the central screw design and a lack of Morse taper. However, this case series shows that this complication is still possible with rTSA designs incorporating a central screw and a peripheral Morse taper design. The decision to proceed with the revision operation depends on the clinical presentation and x-ray.

## Disclaimers:

Funding: No funding was disclosed by the authors.

Conflicts of interest: The authors, their immediate families, and any research foundations with which they are affiliated have not received any financial payments or other benefits from any commercial entity related to the subject of this article.

Patient consent: Obtained.
